# Downclimbing and the evolution of ape forelimb morphologies

**DOI:** 10.1098/rsos.230145

**Published:** 2023-09-06

**Authors:** Luke D. Fannin, Mary S. Joy, Nathaniel J. Dominy, W. Scott McGraw, Jeremy M. DeSilva

**Affiliations:** ^1^ Department of Anthropology, Dartmouth College, Hanover, NH 03755, USA; ^2^ Ecology, Evolution, Environment and Society, Dartmouth College, Hanover, NH 03755, USA; ^3^ Department of Biological Sciences, Dartmouth College, Hanover, NH 03755, USA; ^4^ Department of Anthropology, The Ohio State University, Columbus, OH 43210, USA

**Keywords:** kinematics, descents, ascents, vertical climbing, primates, morphology

## Abstract

The forelimbs of hominoid primates (apes) are decidedly more flexible than those of monkeys, especially at the shoulder, elbow and wrist joints. It is tempting to link the greater mobility of these joints to the functional demands of vertical climbing and below-branch suspension, but field-based kinematic studies have found few differences between chimpanzees and monkeys when comparing forelimb excursion angles during vertical ascent (upclimbing). There is, however, a strong theoretical argument for focusing instead on vertical descent (downclimbing), which motivated us to quantify the effects of climbing directionality on the forelimb kinematics of wild chimpanzees (*Pan troglodytes*) and sooty mangabeys (*Cercocebus atys*). We found that the shoulders and elbows of chimpanzees and sooty mangabeys subtended larger joint angles during bouts of downclimbing, and that the magnitude of this difference was greatest among chimpanzees. Our results cast new light on the functional importance of downclimbing, while also burnishing functional hypotheses that emphasize the role of vertical climbing during the evolution of apes, including the human lineage.

## 1. Introduction

Vertical climbing—defined as the ascent or descent of substrates angled greater than 45° to the horizontal plane [1]—is a widespread behaviour across animal forms, including invertebrates [2,3], squamates [4,5] and mammals [6–10]. Strongly associated with arboreal life, vertical-climbing animals must resist downward slips and the pull of gravity, as falling can result in serious injury, death or predation [7]. It follows that natural selection has favoured anatomical or behavioural traits to mitigate slippage, either by means of claws [6,7], surface adhesion [4] or friction [5]. But with few exceptions, primates are devoid of claws or adhesive capabilities, relying instead on the normal forces generated by their limbs to produce friction grips [6–8].

Vertical climbing is a conspicuous and essential behaviour of hominoid primates, including all great apes [11–14] and some human populations [15,16]. The large body size of most apes, relative to monkeys and other primates, is expected to substantially increase the energetic costs of vertical climbing [17] and risk of falling [18], and it is long hypothesized that the morphologies of hominoid shoulders and elbows evolved to mitigate these twin challenges [19–22]. The retention of these same forelimb traits across early hominin taxa is another topic of enduring debate [23], in part because it suggests that vertical climbing was an essential preadaptation for obligate bipedalism [24–30] (but see [31]).

These discussions have focused almost exclusively on vertical ascent and the functional complexes that reduce the downward torque of gravity by positioning the centre of mass closer to a given support [6]. For instance, extreme dorsiflexion of the ankle—common during chimpanzee ascension [32]—is predicted to improve pedal friction by forcing the forelimbs to subtend large angles, enhancing stability during upward propulsion [6,7]. Captive apes affirm this expectation, exhibiting high degrees of shoulder flexion and elbow extension when climbing flexible ropes [33], yet wild chimpanzees rarely achieve the same joint angles when ascending trees, exhibiting monkey-like forelimb kinematics instead [34,35]. Given these contradictory patterns, some scholars have shifted their attention to postural behaviours, suggesting that the highly mobile forelimbs of apes evolved primarily in response to below-branch, arm-hanging suspensory activities [36–38].

All but ignored in this debate is the challenge of reverse (caudal-first) descent (hereafter, downclimbing). The reason is simple enough—gravitational forces are indifferent to the direction of climbing—but even so, only downclimbing requires controlled braking to counteract gravitational, or passive, acceleration; i.e. eccentric muscular contractions to control the reduction of potential energy and corresponding increases in kinetic energy [39–42]. Studies of chameleons [43], tamarins [42] and humans [44] have shown that steeper arboreal declines reduce vertical force production by the lower-positioned limbs. Consequently, greater support and braking from the higher-positioned limbs may be needed to control descent safely [43]. Downclimbing, then, is expected to increase external loading by the forelimbs on the vertical substrate, which would: (i) generate braking impulse via increasing friction; (ii) improve medio-lateral stability; and (iii) reduce downward toppling moments that compromise safety [45–47].

To exert elevated forces on the vertical substrate, it follows that the shoulders and elbows of primates will subtend larger joint angles during downclimbing [6,7], and that the magnitude of this difference will be greatest at larger body sizes [46]. Thus, differences in forelimb mobility between monkeys and larger-bodied apes are more likely to manifest themselves most strongly during downclimbing. Here, we test this hypothesis by comparing the upper limb kinematics of wild chimpanzees and a species of cercopithecid monkey during vertical climbing.

## 2. Material and methods

### 2.1. Study sites and subjects

We observed chimpanzees (*Pan troglodytes schweinfurthii*; Ngogo community) in Kibale National Park, Uganda during two three-week periods in June 2006 and July–August 2007; and we observed sooty mangabeys (*Cercocebus atys*) in the primary study grid of the Taï Monkey Project in Taï National Park, Côte d'Ivoire for two two-month spans: August–September 2019 and January–March 2022. Defined as a montane or lowland rainforest, respectively, the sites have similar densities of canopy-level trees (540 and 507 ha^−1^, respectively) [48,49] and understory poles/saplings (4290 and 3687 ha^−1^, respectively) [49,50].

Sooty mangabeys are exceptional among cercopithecid monkeys for exhibiting a suite of derived skeletal traits associated with vertical climbing [51,52]. Accordingly, we sought to verify classic monkey–ape morphometric differences in the glenohumeral and humeroulnar joints of our two study species. We accessed osteological collections housed in the Museum of Comparative Zoology, Harvard University and the Department of Anthropology, Ohio State University. We calculated size-standardized measures of glenoid width and depth [53] and olecranon process length [19], following standard procedures with linear caliper measurements.

### 2.2. Video capture and kinematic analysis

We filmed chimpanzees at 14 fps with a Canon GL2 hand-held digital video recorder, targeting adult males, although juveniles and females were also filmed opportunistically. We filmed mangabeys at 30 fps using a tripod-mounted Canon EOS 7D video camera, targeting adults and subadults, although some older juveniles were also recorded opportunistically. Filming distances varied between 5 and 10 m, but every bout of vertical climbing was recorded in lateral view and limited to the first 2–5 m of ascent/descent to minimize angle-induced errors [15].

**Figure 1.**
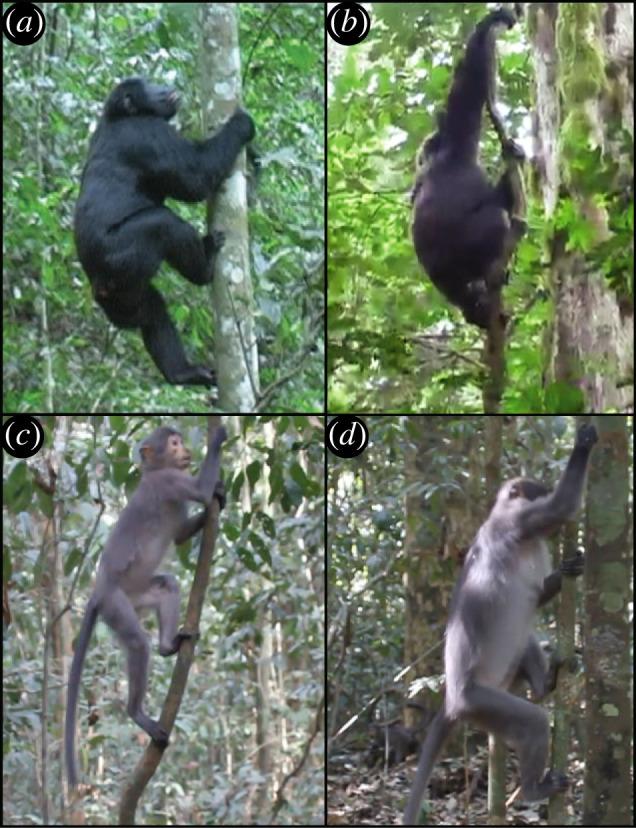
Bouts of vertical climbing in chimpanzees (*a*,*b*) and sooty mangabeys (*c*,*d*). Maximum angles of shoulder flexion and elbow extension were greater during downclimbs (*b*,*d*) compared to upclimbs (*a*,*c*), and the magnitudes of these differences were greatest among chimpanzees. Panels (*a*) and (*b*) taken by J.M.D and panels (*c*) and (*d*) taken by L.D.F.

Following DeSilva [32], we isolated video stills of climbing bouts that depicted the shoulder joint in lateral view at the points of maximum excursion, defined as the largest visible joint angles during shoulder (glenohumeral) flexion and elbow (humeroulnar) extension. Following Levangie & Norkin [54], we defined shoulder flexion as the anterior movement of the humerus in the sagittal plane around a coronal axis passing through the centre of the humeral head, and elbow extension as the posterior movement of the forearm in the sagittal plane around a coronal axis passing through the humeroulnar joint. Shoulder flexion was measured relative to the bole of the tree, which was parallel to the gravitational vector in most videos (figure 1; electronic supplementary material, figure S1). It is also the most functionally relevant reference point for exploring species-level contrasts in shoulder morphology and mobility [53]. Following similar reasoning [36], elbow extension was measured relative to the position of the upper arm. We preferentially targeted the near side of the animal, using three points to define the excursion angles of the shoulder and elbow joints (electronic supplementary material, figure S1). We calculated excursion angles using the manual angle tools in Kinovea v0.8.15 or ImageJ v2.3.0 [55]. We compared angles during vertical ascent and descent intraspecifically (one-tailed tests) and interspecifically (two-tailed tests) using unpooled two-sample *t*-tests in JMP 16 (SAS Institute, Cary, NC, USA).

To assess intra- and inter-observer reliability, we examined a subset of 10 video stills using the protocol of Guatelli-Steinberg *et al*. [56]. Briefly, L.D.F. and M.S.J. measured shoulder and elbow excursion angles from each still, replicating the process three days later to produce a total of 40 measures. We calculated inter- and intra-observer error and used the *irr* package in R (version 4.2.3) to calculate intraclass correlation coefficients (ICCs) [57]. ICCs can be used to evaluate intra- and inter-observer reliability, with values ranging from 0 (no reliability between observers) to 1 (perfect reliability between observers). Average intra-observer measurement error for all joint angles was ±2% (ICCs for both joints = 0.98 for L.D.F. and 0.99 for M.S.J.), whereas average inter-observer error was ±3% for shoulder flexion (ICC: 0.97 (95% CI: 0.89 < ICC < 0.992)) and ±2% for elbow extension (ICC coefficient: 0.98 (95% CI: 0.935 < ICC < 0.995)). Overall, this level of agreement compares favourably to previous kinematic studies of wild primates [32].

Given that substrate diameters could affect forelimb kinematics during climbing [35], we measured the diameter at breast height (DBH; 1.5 m) of most trees associated with climbing bouts. We used the *overlapping* package in R [58] to determine interspecific overlap in the DBH of trees climbed and used regression to assess the potential effects of DBH on shoulder and elbow excursion.

## 3. Results

### 3.1. Intraspecific differences during climbing

**Figure 2.**
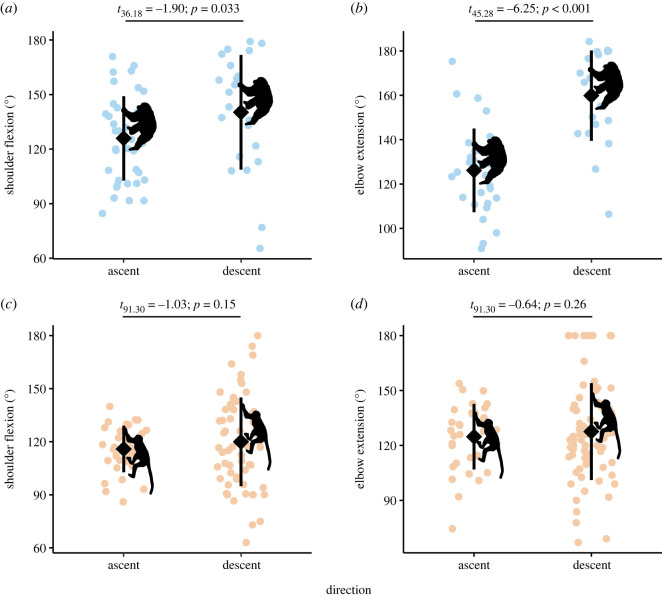
The effect of vertical climbing direction on shoulder flexion and elbow extension in chimpanzees (*a*,*b*) and sooty mangabeys (*c*,*d*). Chimpanzees (light blue) exhibited significantly higher degrees of average shoulder flexion (*a*) and elbow extension (*b*) during vertical descent compared to vertical ascent. By contrast, shoulder flexion (*c*) and elbow extension (*d*) in sooty mangabeys (light orange) were invariant across climbing directions. Diamonds indicate means and vertical bars are ±1 s.d.

Chimpanzee maximum shoulder flexion was 14° greater during bouts of downclimbing (mean: 140 ± 7° (s.e.); *n* = 23 bouts) than upclimbing (mean: 126 ± 4°; *n* = 39 bouts; figure 2*a*), and maximum elbow extension was 34° greater during bouts of downclimbing (mean: 160 ± 3°; *n* = 23 bouts) than upclimbing (mean: 126 ± 4°; *n* = 32 bouts; figure 2*b*). Among mangabeys, such differences were marginal: maximum shoulder flexion was 4° greater during downclimbing (mean: 120 ± 3°; *n* = 63) than upclimbing (mean: 116 ± 3°; *n* = 32; figure 2*c*), and maximum elbow extension was 3° greater during bouts of downclimbing (mean: 128 ± 3°; *n* = 71 bouts) than upclimbing (mean: 125 ± 3°; *n* = 34 bouts; figure 2*d*).

### 3.2. Interspecific differences during climbing

**Figure 3.**
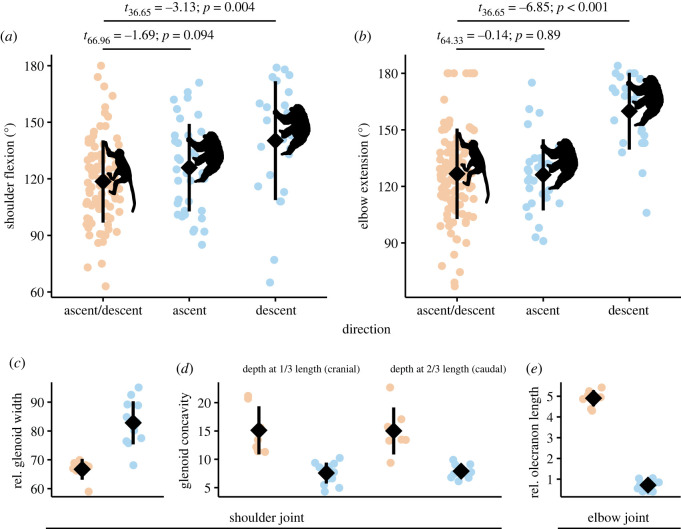
Forelimb vertical climbing kinematic (*a*,*b*) and morphological (*c–e*) contrasts between chimpanzees (light blue) and sooty mangabeys (light orange). Chimpanzees exhibited significantly greater degrees of shoulder flexion (*a*) and elbow extension (*b*) than sooty mangabeys during vertical descent, but not vertical ascent. Sooty mangabeys had relatively narrower (*c*) and craniocaudally curved (*d*) glenoid cavities, but larger olecranon processes than chimpanzees (*e*). Diamonds indicate means and vertical bars are ±1 s.d. See electronic supplementary material for definitions of size-standard measurements.

To simplify interspecific comparisons, we compared joint angles during up- and downclimbing by mangabeys and pooled the datasets based on their homogeneity (figure 2*c*,*d*). During ascent, the degree of shoulder flexion and elbow extension was similar between the two species (figure 3*a*,*b*), but during descent, the degree of shoulder flexion and elbow extension differed greatly, with chimpanzees expressing mean joint angles that were 21° and 33° greater, respectively, than those of mangabeys (figure 3*a*,*b*).

### 3.3. Comparative morphometrics

Relative to mangabeys, we found that the glenoid cavities of chimpanzees are wider, uniformly shallower, and more ovate (figure 3*c*,*d*), and that their olecranon processes are shorter (figure 3*e*), which is consistent with expected ape–monkey differences in forelimb functional morphology.

### 3.4. Diameter at breast height and joint angles

The DBH of climbed trees differed between the two species—chimpanzees climbed wider trees (mean DBH = 14 ± 8 cm; *n* = 166) than those used by mangabeys (mean DBH = 6 ± 3 cm; *n* = 259), but observed DBHs overlapped by 24%. We found no statistical effect of DBH on shoulder flexion or elbow extension among chimpanzees (electronic supplementary material, figure S2). Variation in DBH explained approximately 10% of variation in mangabey shoulder flexion, but there was no statistical relationship with elbow extension (electronic supplementary material, figure S2).

## 4. Discussion

We found that the maximum angles of shoulder flexion and elbow extension of chimpanzees and sooty mangabeys were largest during downclimbing, and that the magnitude of these differences was greatest among chimpanzees. The significance of our findings is twofold. First, our data corroborate conclusions drawn from observational categorical data: chimpanzees and cercopithecid monkeys use their forelimbs in kinematically similar ways during upclimbing [34–37]. Second, our findings extend classic [6,7] and more recent [39–41] theoretical discussions of vertical climbing by casting new light on the functional importance of downclimbing during ape evolution.

The evolutionary milestones of ape forelimb mobility are attested by several fossil taxa. For instance, the early Miocene ape *Morotopithecus* (approx. 21 Ma) possessed an ape-like glenoid cavity, which is interpreted as evidence of ‘forelimb suspensory and forelimb dominated climbing behaviours, including vertical *ascension*’ (emphasis ours) [53]. The earliest evidence of olecranon reduction emerged later in *Danuvius* (12 Ma) and *Hispanopithecus* (10 Ma), and is fully modern with the appearance of *Oreopithecus* (7 Ma) [59,60]. Authors have interpreted this aspect of elbow morphology as an ‘unmistakable hallmark of below-branch or suspensory behaviour' [60]; or evidence of ‘eclectic climbing and below-branch suspensory behaviours’ [59]. We do not dispute the essential role of these positional behaviours, but we would argue that these same morphologies also speak loudly to the critical importance of controlled downclimbing.

Improved downclimbing among Miocene apes can be viewed as a by-product, or ‘spandrel' [61], of selection favouring below-branch suspension in some large-bodied lineages [62]. But it also raises the possibility of changing arboreal conditions, such as increasing canopy stratification or rugosity [63–65], which would favour more frequent movement in the vertical dimension. Another possibility pivots around increasing terrestrial behaviours. Indeed, there is evidence of Miocene apes exploiting non-arboreal foods, such as fallen fruits [66] as well as aquatic plants and underground storage organs [67]; and further, some habitat reconstructions would appear to favour frequent downclimbing to traverse heterogeneous swamp-woodlands [68] or wooded grasslands [69]. Dual use of terrestrial and arboreal milieus, mediated by frequent downclimbs, may also explain the retention of ancestral, ape-like traits in the forelimbs of early fossil hominins such as *Sahelanthropus* [70], *Ardipithecus* [71] and *Australopithecus* [72]. This perspective begins to reconcile competing hypotheses focused on either postural or locomotor behaviours [36], as downclimbing is necessary to connect both.

Contrary to published predictions [34,35], we found mixed evidence for DBH-mediated effects on joint angles during climbing, but these results are partially underpowered with only 12 DBH associations for chimpanzees. Thus, our limited sample size was insufficient for testing the hypothesized (positive) effect of larger DBH on chimpanzee elbow extension during vertical climbing. Moving forward, it would be instructive to explore more systematically if and how substrate properties (e.g. size, angle, surface texture) affect the forelimb kinematics of downclimbing. Another limitation of our study concerns climbing speed, which we were unable to measure, and its potential effects on the kinematics of downclimbing. Greater travel speed can affect forelimb protraction during knuckle-based quadrupedalism [73], but there is little evidence of it affecting shoulder or elbow excursions during upclimbing [33]. Still, passive acceleration due to gravity could make downclimbing faster and inherently less stable [39], factors that could favour a braking impulse and greater forelimb excursion angles. In such cases, the velocity of descent is expected to be lower than that of ascent, as reported for some primates that descend trees headfirst [74]; but see [42].

Species inviting future comparative study include the larger-bodied forest papionins of West Africa. Wild mandrills (*Mandrillus sphinx*), for example, attain masses up to 36 kg [75] and it is an open question whether they descend trees in a monkey- or ape-like fashion. Investigating downclimbing in olive baboons (*Papio anubis*) would also be informative, since they too can attain large masses (approx. 40 kg) while upclimbing in a manner like chimpanzees [11]. Lastly, our functional predictions at the outset drew on deductive logic, arguing that downclimbing increases energetic costs and the risk of falling, factors that are expected to incur relatively high fitness costs [46]. This premise could be further tested among human populations that regularly climb vertical surfaces [76], including large-diameter trees [77].

## Data Availability

Data are accessible in the Dryad Digital Repository: https://doi.org/10.5061/dryad.hqbzkh1m8 [78]. Supplementary figures are provided in the electronic supplementary material [79].
